# Imaging assessment of children presenting with suspected or known juvenile idiopathic arthritis: ESSR-ESPR points to consider

**DOI:** 10.1007/s00330-020-06807-8

**Published:** 2020-05-12

**Authors:** Robert Hemke, Nele Herregods, Jacob L. Jaremko, Gunnar Åström, Derk Avenarius, Fabio Becce, Dennis K. Bielecki, Mikael Boesen, Danoob Dalili, Chiara Giraudo, Kay-Geert Hermann, Paul Humphries, Amanda Isaac, Anne Grethe Jurik, Andrea S. Klauser, Ola Kvist, Frederiek Laloo, Mario Maas, Adam Mester, Edwin Oei, Amaka C. Offiah, Patrick Omoumi, Olympia Papakonstantinou, Athena Plagou, Susan Shelmerdine, Paolo Simoni, Iwona Sudoł-Szopińska, Laura Tanturri de Horatio, James Teh, Lennart Jans, Karen Rosendahl

**Affiliations:** 1 Department of Radiology and Nuclear Medicine, Amsterdam University Medical Centers, Amsterdam Movement Sciences, Academic Medical Center, University of Amsterdam, Meibergdreef 9, 1105 AZ Amsterdam, The Netherlands; 2 Department of Radiology and Medical Imaging, Ghent University Hospital, Ghent, Belgium; 3 Department of Radiology and Diagnostic Imaging, Faculty of Medicine and Dentistry, University of Alberta, Edmonton, Canada; 4 Department of Radiology, Uppsala University, Uppsala, Sweden; 5 Department of Radiology, University Hospital of North Norway, Tromsø, Norway; 6 Department of Diagnostic and Interventional Radiology, Lausanne University Hospital, Lausanne, Switzerland; 7 Department of Diagnostic Imaging, Kings College Hospital, London, UK; 8 Department of Radiology, Bispebjerg and Frederiksberg Hospital, Copenhagen, Denmark; 9 Department of Radiology, Chelsea and Westminster Hospital NHS Foundation Trust, London, UK; 10 Radiology Institute, Department of Medicine – DIMED, Padova University, Padua, Italy; 11 Department of Radiology, University Hospital Charité, Berlin, Germany; 12 Department of Radiology, Great Ormond Street Hospital, London, UK; 13 Department of Radiology, Guy’s & St Thomas Hospitals, London, UK; 14 Department of Radiology, Aarhus University Hospital, Aarhus, Denmark; 15 Department of Radiology, Medical University of Innsbruck, Innsbruck, Austria; 16 Department of Paediatric Radiology, Karolinska University Hospital, Stockholm, Sweden; 17 Department of Radiology, National Institute of Rheumatology and Physiotherapy, Budapest, Hungary; 18 Department of Radiology and Nuclear Medicine, Erasmus University Medical Center (Erasmus MC), Rotterdam, The Netherlands; 19 Academic Unit of Child Health, University of Sheffield, Western Bank, Sheffield, UK; 20 Department of Radiology, “Attikon” Hospital, National University of Athens, Athens, Greece; 21 Private Radiological Institution, Athens, Greece; 22 Department of Radiology, Reine Fabiola Children’s University Hospital of Bruxelles, University of Bruxelles, Brussels, Belgium; 23 Department of Radiology, National Institute of Geriatrics, Rheumatology and Rehabilitation and Department of Medical Imaging, Medical University of Warsaw, Warsaw, Poland; 24 Department of Diagnostic Imaging, Bambino Gesù Children’s Hospital, Rome, Italy; 25 Department of Radiology, Nuffield Orthopaedic Centre, Oxford University Hospitals NHS Trust, Oxford, UK

**Keywords:** Diagnostic imaging, Juvenile idiopathic arthritis, Magnetic resonance imaging, Conventional radiography, Ultrasound computed tomography

## Abstract

**Abstract:**

Juvenile idiopathic arthritis (JIA) is the most common paediatric rheumatic disease. It represents a group of heterogenous inflammatory disorders with unknown origin and is a diagnosis of exclusion in which imaging plays an important role. JIA is defined as arthritis of one or more joints that begins before the age of 16 years, persists for more than 6 weeks and is of unknown aetiology and pathophysiology. The clinical goal is early suppression of inflammation to prevent irreversible joint damage which has shifted the emphasis from detecting established joint damage to proactively detecting inflammatory change. This drives the need for imaging techniques that are more sensitive than conventional radiography in the evaluation of inflammatory processes as well as early osteochondral change. Physical examination has limited reliability, even if performed by an experienced clinician, emphasising the importance of imaging to aid in clinical decision-making. On behalf of the European Society of Musculoskeletal Radiology (ESSR) arthritis subcommittee and the European Society of Paediatric Radiology (ESPR) musculoskeletal imaging taskforce, based on literature review and/or expert opinion, we discuss paediatric-specific imaging characteristics of the most commonly involved, in literature best documented and clinically important joints in JIA, namely the temporomandibular joints (TMJs), spine, sacroiliac (SI) joints, wrists, hips and knees, followed by a clinically applicable point to consider for each joint. We will also touch upon controversies in the current literature that remain to be resolved with ongoing research.

**Key Points:**

• *Juvenile idiopathic arthritis (JIA) is the most common chronic paediatric rheumatic disease and, in JIA imaging, is increasingly important to aid in clinical decision-making.*

• *Conventional radiographs have a lower sensitivity and specificity for detection of disease activity and early destructive change, as compared to MRI or ultrasound. Nonetheless, radiography remains important, particularly in narrowing the differential diagnosis and evaluating growth disturbances.*

• *Mainly in peripheral joints, ultrasound can be helpful for assessment of inflammation and guiding joint injections. In JIA, MRI is the most validated technique. MRI should be considered as the modality of choice to assess the axial skeleton or where the clinical presentation overlaps with JIA.*

**Electronic supplementary material:**

The online version of this article (10.1007/s00330-020-06807-8) contains supplementary material, which is available to authorized users.

## Paediatric-specific items per joint

### Axial joints

#### Temporomandibular joints

Temporomandibular joint (TMJ) involvement is common in children with juvenile idiopathic arthritis (JIA), and it is often present early in the disease [1]. It has been implicated in 40–87% of JIA patients on magnetic resonance imaging (MRI) [2–6]. Similar to the involvement of other axial joints, TMJ involvement in JIA is difficult to detect clinically [7].

**Fig. 1:**
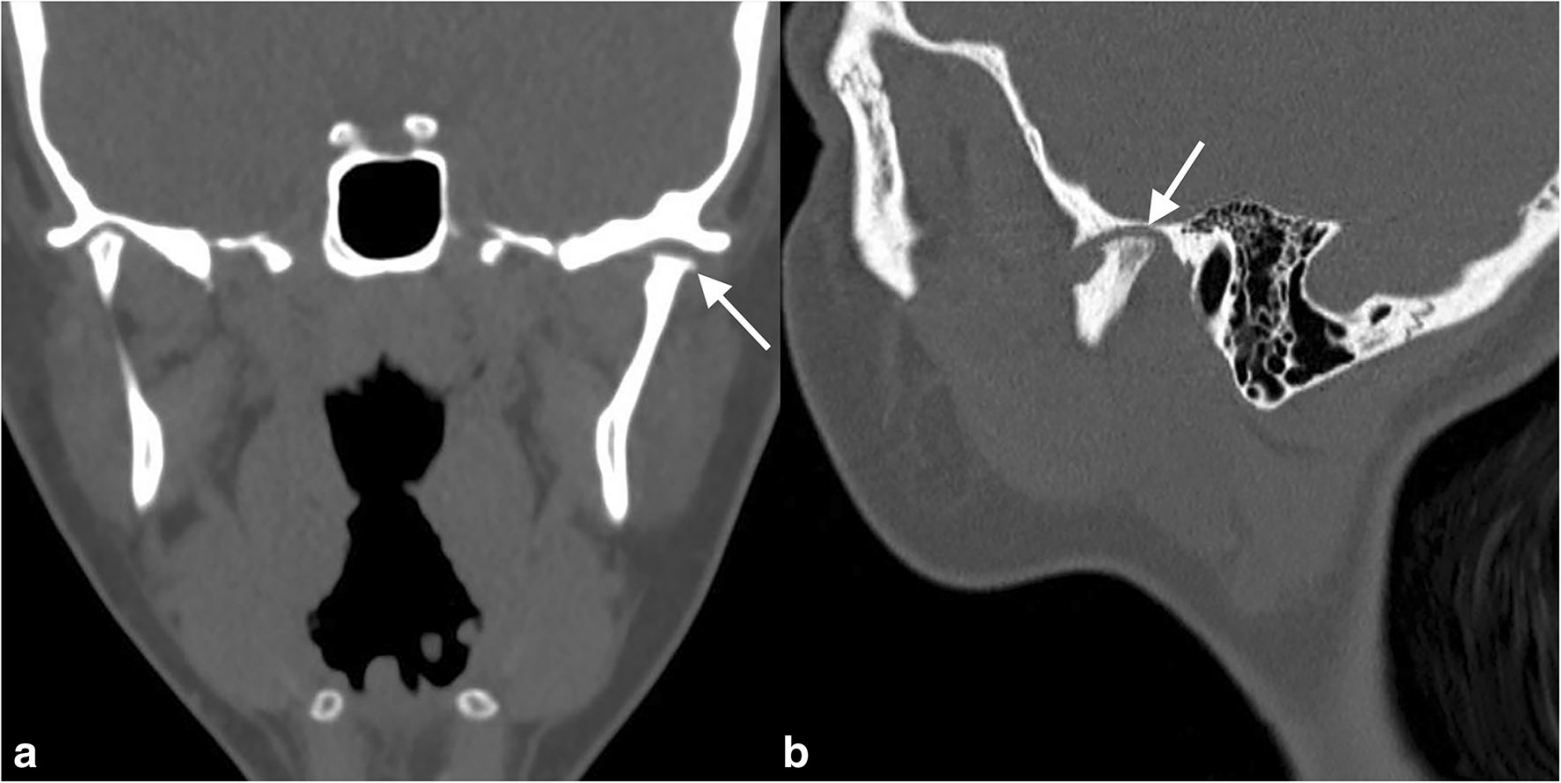
An 18-year-old girl with long-standing TMJ arthritis showing chronic condylar bony abnormalities of the left temporomandibular joint including flattening of the temporal fossa and the mandibular condyle (arrows) on (**a**) coronal and (**b**) sagittal CT images

The main growth centre of the mandible is located in the condyle, and mandibular growth is therefore vulnerable to arthritic changes [8]. Early detection and treatment of TMJ arthritis is important to preserve mobility and to prevent growth abnormalities and deformities which have been found to be associated with impaired health-related quality of life [9]. Conventional radiography and cone beam computed tomography (CT) are used to detect condylar bony abnormalities (Fig. 1). Compared to conventional radiography, CT and cone beam CT avoid difficulties of superimposition and offer unsurpassed resolution of cortical surfaces, but soft tissue changes such as those related to the disc and joint capsule as well as bone marrow oedema cannot accurately be assessed [9, 10].

Ultrasound (US) could potentially detect both osteochondral and soft tissue changes. It is, however, not practical for the assessment of axial joint arthritis. US was found to moderately correlate with contrast-enhanced MRI in the detection of TMJ involvement in children with JIA [11, 12]. The usefulness of ultrasound for the TMJ is limited due to the complex nature of this joint [13]. There are no accepted US-based normative values for synovial thickness (Table 1), and a valid US scoring system for the TMJ is lacking (Table 2).

**Table 1:** Joint-specific paediatric normal references by modality

Joint	Radiography	Ultrasound	MRI
TMJ	NA	NA	Kottke et al [15]
Spine	NA	NA	NA
SI joint	NA	NA	Chauvin et al [30]
Wrist	Greulich and Pyle [31]	Rosendahl et al [32] Collado et al [33] Roth et al [34]	Ording Muller et al [35] Avenarius et al [36]
Hip	NA	Rohrschneider et al [37] Robben [38]	NA
Knee	NA	Collado et al [33] Roth et al [34] Keshava et al [39] Spannow et al [40]	Hemke et al [41]

**Table 2:** Joint-specific scoring systems for evaluating inflammatory and/or destructive changes by modality

Joint	Radiography	Ultrasound	MRI
TMJ	NA	NA	Koos et al [42] Vaid et al [21]
Spine	NA	NA	NA
SI joint	NA	NA	Weiss et al [43] Herregods et al [44]
Wrist	Adapted Sharp/van der Heijde [46] Poznanski score [47]	NA	Malattia et al [45] Damasio et al [49]
Hip	Shelmerdine et al [50] Bertamino et al [51]	NA	NA
Knee	NA	CARRA JIA Ultrasound Workgroup [52]	Juvenile Arthritis MRI Score (JAMRIS) [53, 54]

**Fig. 2:**
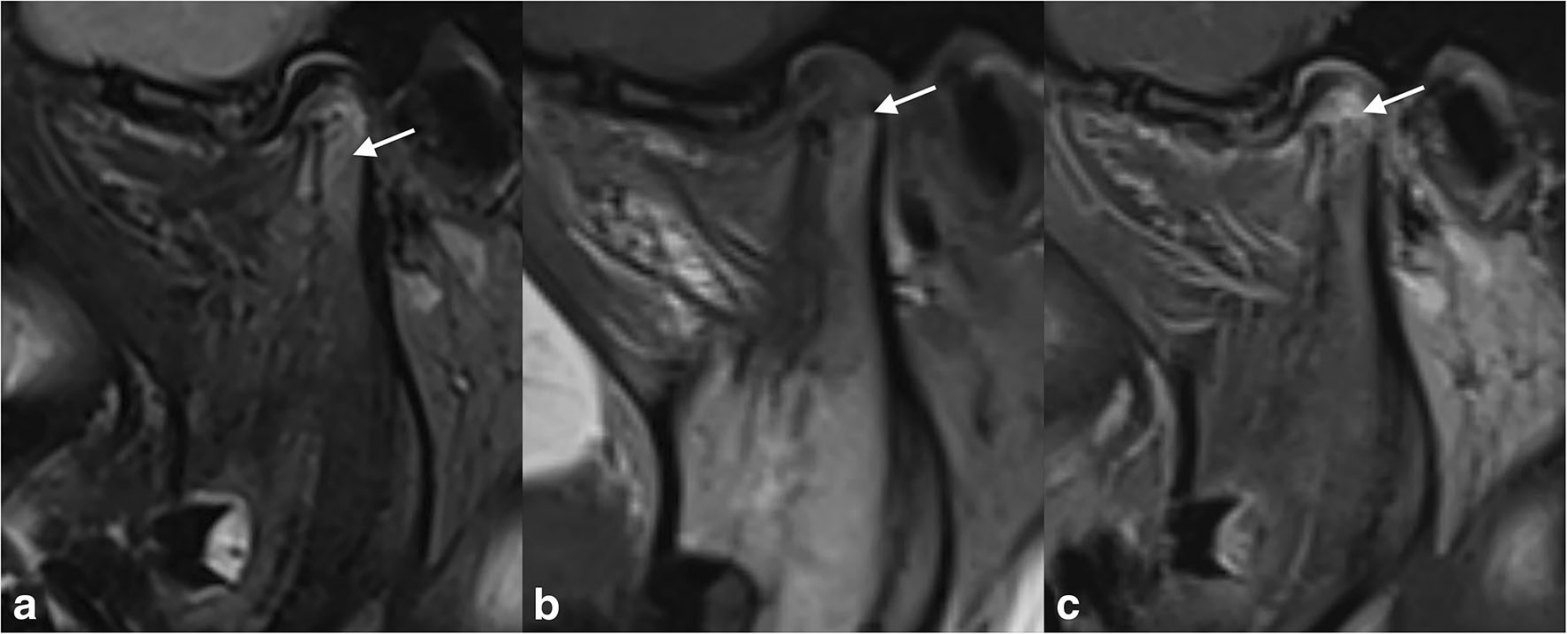
A 13-year-old girl with JIA and active TMJ arthritis. MRI of the TMJ with (**a**) a sagittal oblique T2-weighted fat-saturated image showing bone marrow oedema (hyper-intense signal on T2-weighted images (arrow)) in the TMJ condyle, (**b**) a sagittal oblique T1-weigted image showing bone marrow oedema (hypo-intense signal on T1-weighted images) and condylar flattening (arrow) and (**c**) a sagittal oblique T1-weighted fat-saturated post-Gd image showing joint and condylar enhancement (arrow)

MRI is the modality of choice for the assessment of TMJ arthritis as it can detect acute and early inflammatory changes consisting of joint effusion, synovial enhancement/thickening and bone marrow oedema, as well as chronic changes including erosions, osseous deformity, new bone formation and disc abnormalities [14]. Small dots or lines of high signal intensity on T2-weighted sequences within the joint recesses are considered physiological joint fluid [15]. Synovial thickness is difficult to measure due to rapid diffusion of contrast to the synovial fluid, but comparing post-contrast T1-weighted fat-saturated images to pre-contrast T2-weighted fat-saturated images which demonstrates the extent of joint effusion can be helpful [9, 16]. Figure 2 depicts an example of active TMJ arthritis on MRI. For optimal evaluation of the TMJ, an MRI protocol preferably includes sequences with open and closed mouth. For evaluating the disc position and function in relation to the condyle, open-mouth views are valuable when compared with closed-mouth views [17]. The condyle morphology is best evaluated with a closed-mouth view [17]. Some MRI scoring systems for TMJ evaluation in JIA are available (Table 2). An example of an MRI protocol for the TMJ in JIA is given in Supplementary File 1.

##### Point to consider

TMJ MRI could be performed in patients suspected clinically of TMJ involvement, with fluid-sensitive, closed and open mouth, and potentially, post-gadolinium sequences.

##### Rationale

Detection of TMJ involvement allows earlier treatment which may reduce growth deformity and TMJ dysfunction. Closed and open mouth imaging may demonstrate alternative mechanical aetiologies for findings and can help in understanding the functional limitations of the joint, as well as the impact of JIA on the TMJ disc.

##### Controversies/future developments

(a) Given that TMJ arthropathy is often clinically silent, should patients with JIA have screening TMJ MRI? (b) Is gadolinium necessary to depict inflammation, or does fluid-sensitive imaging suffice?

#### Spine

In children with JIA and spine involvement, the cervical spine is most frequently involved. Up to 65% of JIA patients have symptoms of the cervical spine [18–20]. There is also an association between TMJ and cervical spine arthritis [21]. The atlanto-occipital and atlanto-axial joints are synovial joints and are prone to rheumatoid inflammation [22]. Cervical spine arthritis can sometimes follow a severe course, resulting in morphological change and functional impairment when left untreated [23]. The clinical signs and symptoms in children with spinal involvement differ from those in adults [24]. Since inflammatory back pain being less prominent in children, sacroiliac (SI) joint arthritis/enthesitis, involved infrequently, and hip and peripheral joint arthritis/enthesitis are commonly seen in children with enthesitis-related arthritis (ERA), and inflammatory abnormalities involving the spine can be missed in children [25]. As with TMJ arthritis, relatively minor subjective complaints are often associated with extensive imaging abnormalities [26]; therefore, evaluating the whole spine can be helpful to increase diagnostic accuracy. Thoracic and lumbar spinal inflammatory lesions, which are relatively common in adults, are rare in children [24, 25], especially in the early phase of the disease and in the absence of sacroiliitis [24].

Radiography is useful for assessing malalignment, functional impairment, growth disturbances or morphological bony changes [27, 28]. Apophyseal joint ankylosis, anterior atlanto-axial subluxation and atlantoaxial impaction are serious complications of rheumatoid arthritis, but these are rare in children [29]. Atlanto-axial diastases may be normal in paediatric patients; therefore, dynamic radiographic views must be interpreted with caution. Radiography is not sensitive for detecting early joint changes [55].

Concerning the cervical joints, there are no published studies on the use of US in JIA.

Contrast-enhanced MRI is the modality of choice for detecting early, often subclinical cervical spine arthritis, with joint effusion, enhancing thickened synovium, and bone marrow oedema. MRI can also evaluate late stage changes such as erosions, dens deformation, subluxations, joint ankylosis and neural compression [23, 26, 56, 57]. In adults, bone marrow oedema is considered a predictor for erosions [58]. In adults, the Assessment in Spondyloarthritis International Society (ASAS) identified features that could indicate a positive spinal MRI for spondyloarthritis (SpA) [59]. However, these definitions developed for adults have not yet been validated in children, with no endorsed scoring system available for MRI evaluation of arthritis of the spine in children [60]. An example of an MRI protocol is given in Supplementary File 1.

##### Point to consider

Radiography of the spine is still suggested in JIA patients with clinical involvement of the spine, but in terms of diagnostic accuracy in early disease and radiation protection, MRI of the whole spine can be considered at baseline.

##### Rationale

Ultrasound of the SI and spinal joints is neither practical nor reliable. Radiographs may depict late structural damage and syndesmophytes, whilst identifying anatomic variants and abnormalities which may give alternative mechanical explanations for pathology. MRI depicts bony and soft tissue features of both early and chronic diseases and can both quantify disease burden and monitor treatment effect.

##### Controversies/future developments

(a) Can low-dose CT replace or supplement radiography in order to depict structural bony changes at an earlier stage? (b) Should screening MR images of the cervical spine be included in a TMJ arthritis protocol?

#### Sacroiliac joints

The SI joints are affected in approximately 30% of children with the ERA subtype of JIA. Sacroiliitis is usually not seen in the early course of the disease; children typically first present with enthesitis and lower extremity peripheral arthritis prior to developing SI joint involvement. Despite this, early identification of sacroiliitis is crucial, as treatment options are not only different than those for peripheral juvenile SpA, but there is also markedly increased long-term disability too. Clinical assessment of the SI joints is difficult, with non-specific and subjective symptoms that may occur rather late in the disease course.

Radiographs have limited value in screening for sacroiliitis in children and result in a significant proportion of both false negative and positive findings compared to MRI [ 61–64]. As discussed earlier, the usefulness of US in axial joints is limited.

**Fig. 3:**
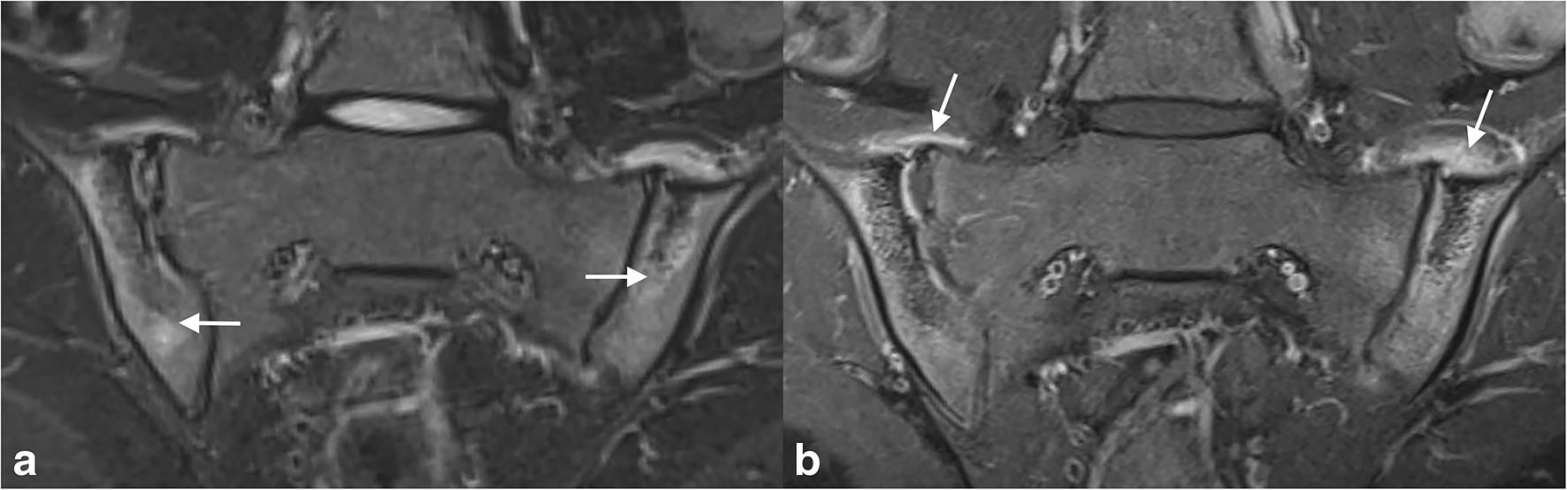
A 16-year-old boy with JIA, active sacroiliitis and an MRI showing (**a**) a coronal oblique STIR image showing bone marrow oedema (arrow) in, predominantly, the iliac side of the sacroiliac joints and capsulitis and (**b**) a coronal oblique T1-weigted fat-saturated post-Gd image showing bone marrow enhancement, joint enhancement and capsulitis (arrow). In this case, capsulitis can be seen as hyper-intense T2-weighted signal with enhancement at the T1-weighted fat-saturated post-Gd image at the cranial site of the sacroiliac joints

MRI is the imaging modality of choice for detecting early inflammatory change of the SI joints. Active features of sacroiliitis can include bone marrow oedema, enthesitis and capsulitis/synovitis (Fig. 3). Features of structural damage include erosions, fatty deposition, sclerosis and ankylosis. According to the ASAS definition of sacroiliitis suggestive of SpA in adults, bone marrow oedema must be periarticular in location [65]. Although water-sensitive sequences alone are highly sensitive for the detection of active sacroiliitis, contrast-enhanced (fat-saturated) T1-weighted sequences may be helpful to differentiate joint fluid from synovitis [4, 66, 67]. See Supplementary File 1 for an example of an MRI protocol. In contrast to sacroiliitis in adults, bone marrow oedema is highly specific for juvenile SpA and is less dependent on other features of SpA for imaging diagnosis. The hips are commonly affected in ERA; therefore, they should be included in MRI of SI joints [64]. In adults, there are scoring systems, of which the Spondyloarthritis Research Consortium of Canada (SPARCC) scoring system is most widely accepted. The early studies in children are promising and describe good feasibility and reliability of the SPARCC scoring systems; however, these are not yet widely used and some adaptations may be necessary [43–45] (Table 2). Developing reliable paediatric-specific definition for sacroiliitis is a difficult task currently undergoing active study [44, 68]. A paediatric-specific scoring system is being developed by the Outcome Measures in Rheumatology Clinical Trials (OMERACT) MRI in JIA working group [60].

##### Point to consider

Children with suspected axial SpA could have MRI performed to include SI joints and hips. Including screening MR images of the whole spine is preferred.

##### Rationale

Radiography has poor sensitivity and specificity for detecting sacroiliitis. Performing MRI of only a limited area of the SI joints may miss clinically silent disease of the hips and spine which adds to the understanding of overall disease burden and may affect prognosis.

##### Controversies/future developments

(a) In growing children, it can be difficult to differentiate normal variants from pathology. How should we, therefore, formally define a *positive* scan in each region, particularly when normal standards are lacking? (b) Are there any situations in which gadolinium is crucial for MRI of the SI joints in children?

### Peripheral joints

#### Wrist

Wrist involvement in JIA occurs in about 25% of patients, increasing to 40% after 5 years of disease [69]. In JIA, early involvement of the wrist, distal small joint arthritis and a symmetric arthritis are poor prognostic factors [55]. Since early recognition and proper treatment can improve clinical outcome [70], imaging plays an important role in JIA patients with hand and wrist involvement.

Conventional radiography has been considered the basis to identify growth abnormalities and late destructive change [71]. Also, cartilage loss is hard to evaluate in growing children. Several scoring systems for evaluating structural damage in children with JIA and hand/wrist involvement exist, of which an adapted version of the Sharp/van der Heijde score has been shown to be both reliable and valid for progressive change [46] (Table 2). The Poznanski index is a useful measure of late change [47]; particularly in younger children, bone damage can appear as squaring or deformity of the carpal bones and epiphyses rather than as true erosive change [71]. Conventional radiography is superior to MRI in this regard [55, 72].

US is helpful for the assessment of inflammation as well as for guidance in joint injections. It has been shown to reliably detect synovitis, tenosynovitis, cartilage damage and bone erosions in the wrist and metacarpal joints of JIA patients [73]. Currently, no agreed scoring systems exist; however, this is work-in-progress [71] (Table 2). Typically, there is a thickened, hyperaemic synovial membrane and a joint effusion. Some standards for synovial thickness and the amount of joint fluid exist for the wrist (Table 1). Definitions of age-dependent ultrasonographic anatomy and standardised approach for ultrasound in children have been suggested [33, 34].

**Fig. 4:**
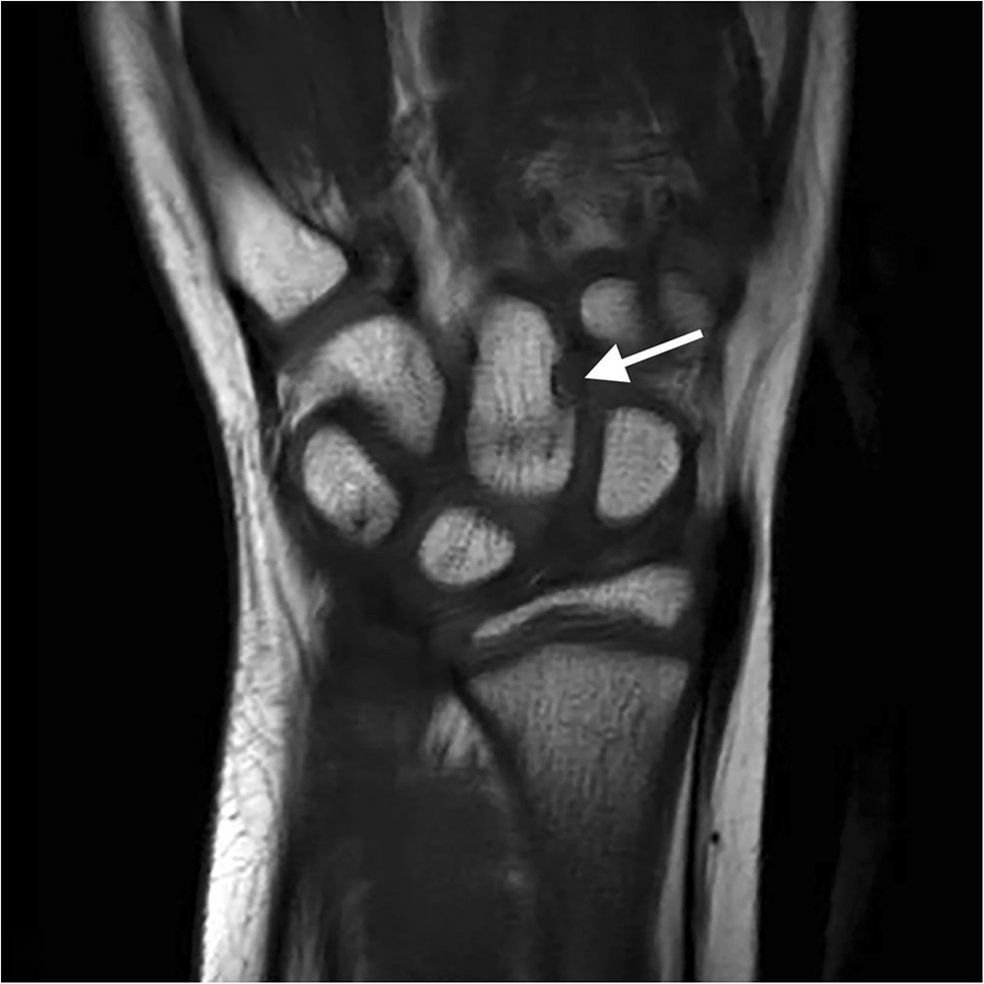
A 10-year-old healthy girl with a coronal T1-weighted image showing a bony depression on the radial side of the capitate (arrow). This is a normal depression that can be seen in the carpal bones of growing children and should not be mistaken for pathology (erosive disease)

MRI is the most validated method for assessment of inflammation, showing synovitis, tenosynovitis and effusion [71]. It also shows bone marrow oedema and late destructive change [74]. There are several sequences which are helpful for the assessment of disease activity and structural change [75]. This includes pre- and post-contrast fat-saturated images (in the same plane) to differentiate an inflamed synovium from joint effusion [76] and a field of view including the distal radio-ulnar joints and the metacarpophalangeal joints [60, 77] (see also Supplementary File 1 for an example of an MRI protocol). The development of a MRI scoring system was initially based on the OMERACT Rheumatoid Arthritis MRI Scoring (RAMRIS) system for adults [78]. Malattia and colleagues [48] developed the first paediatric-targeted MRI scoring system. During the following years, an international effort called Health-e-Child published a revised version [49] and suggested an extension of the field of view [77] (Table 2). It is important to be aware of the high prevalence of normal variants (bony depressions (Fig. 4), bone marrow lesion–like changes and joint fluid) as this may mimic pathology in the scope of JIA [35, 36] (Table 1).

##### Point to consider

Routine radiographs of the wrists/hands are recommended at diagnosis and follow-up of JIA patients with wrist/hand involvement and could be performed alongside MRI or ultrasound.

##### Rationale

It can be hard to differentiate normal bony depressions from erosions in wrists of JIA patients. Growth disturbances of the wrist and periarticular osteoporosis in longer standing JIA are probably a more consistent hallmark of destructive change, which can be more reliably evaluated on conventional radiographs.

##### Controversies/future developments

(1) Will a dedicated additional cartilage sequence help to differentiate normal variants from pathologic erosive change in the JIA wrist with more certainty? (2) Can dynamic contrast-enhanced MRI help to differentiate active from inactive inflammation from physiological increased perfusion in the joint tissue due to growth?

#### Hip

The hip is affected in around 20–50% of the children with JIA [79, 80] and can cause irreversible destructive change within 5 years of diagnosis [81].

Imaging findings are those of inflammation (synovitis, tendinitis and bursitis) and effusions before peri-articular bony changes (bone marrow oedema) [82]. Further disease progression may lead to growth disturbances as well as destruction of cartilage and bone. Growth disturbances are best imaged radiographically. The only child-specific scoring systems available are those of Bertamino et al [51] and Shelmerdine et al [50] (Table 2).

Normal US reference values for synovial thickness and the presence of visible joint fluid were published decades ago [37, 38] (Table 1). The European Society of Musculoskeletal Radiology (ESSR) provides a free online guide with anatomical correlation and ultrasound features of the hip joint [83]. However, age-related variations in thickness of cartilage, appearance of ossification centres and normal epiphyseal and metaphyseal vessels can mimic pathology [82]. In cases of inflammation, there is a thickened, often villous and hyperaemic synovium and an effusion. Validated US scoring systems for the JIA hip are lacking (Table 2).

**Fig. 5:**
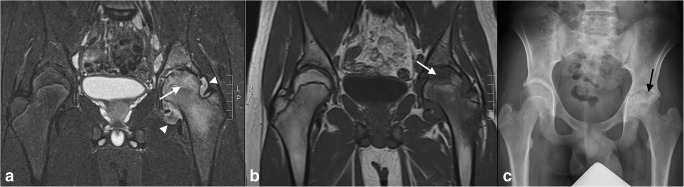
A 15-year-old boy with juvenile idiopathic arthritis and hip involvement with (**a**) a coronal T2-weighted fat-saturated image showing synovial thickening (arrow heads) in the left hip with extensive bone marrow oedema in the femoral head (arrow), (**b**) a coronal T1-weigted image showing irregular cortical linings in the scope of erosive changes (arrow) and (**c**) the corresponding X-ray showing joint space narrowing and cortical irregularities/erosive change in the femoral head (arrow)

MRI is the only modality that can assess both the soft tissue and bone marrow changes seen in JIA [82] (Fig. 5). MRI sequences will usually include one T1-weighted sequence (non-fat–saturated, to assess for appropriate bone marrow fatty conversion), a water-sensitive sequence (to assess for bone marrow oedema and joint effusion) and pre- and post-contrast fat-saturated T1-weighted sequences (to assess for synovial enhancement and thickening). Timing of post-contrast images should be standardised [76, 84]. An example of an MRI protocol is given in Supplementary File 1. Currently, a validated MRI scoring system for the hips has not been established (Table 2). Porter-Young et al [85] have shown the most reliable MRI parameters on which a scoring system might be based.

##### Point to consider

Routine radiographs of the hips could be performed at presentation and follow-up of JIA patients with hip involvement. MRI could be considered at baseline and, potentially, also at follow-up when ultrasound is equivocal.

##### Rationale

Radiographs are important for the assessment of growth abnormalities, and ultrasound is easy to use for the assessment of active inflammation in children. When findings are equivocal, an MRI, preferably with gadolinium, could be performed to confirm the diagnosis and for narrowing the differential diagnoses.

##### Controversies/future developments

Will a dedicated additional cartilage sequence be helpful in the detection of early destructive change?

#### Knee

The knee joint is clinically the most commonly affected joint in JIA [69].

Radiography remains important, particularly in narrowing the differential diagnosis and in establishing a baseline for follow-up. It can provide information on growth disturbances [55, 86]. Because of the availability of more effective treatment options and the relatively large amount of epiphyseal cartilage in knees of growing children, bone erosions in knee joints in JIA patients are relatively rare.

US plays an important role in differential diagnosis and can be useful for treatment monitoring as well as for guiding joint injections [52, 87]. Knee US has some limitation. The central recess, whilst being the location most commonly affected by synovitis in the knee, is difficult to evaluate sonographically [88]. Recently, a paediatric-specific US scoring system for the knee has been proposed by the Childhood Arthritis and Rheumatology Research Alliance (CARRA) JIA Ultrasound Workgroup [52].

MRI is the preferred imaging modality for the assessment of inflammatory and destructive changes in JIA patients with knee involvement. The main imaging features include synovial thickening, joint effusion and bone marrow oedema. Although relatively rare, cartilage loss and bone erosion may be observed. Synovitis is the principal pathological process in JIA, and its presence in the knee is associated with the clinical onset of JIA [89]. Therefore, pre- and post-contrast sequences with standardised timing of post-contrast images are warranted to accurately evaluate synovitis in the knee joint [76, 84, 90]. An example of an MRI protocol for the knee in JIA is given in Supplementary File 1. In recent years, a paediatric-specific MRI scoring system for the knee has been developed and validated (the Juvenile Arthritis MRI Scoring (JAMRIS)) [53, 54] (Table 2). MRI of healthy children may show an enhancing synovial membrane (< 2 mm), some joint fluid and, in some cases, high-signal intensity bone marrow changes in the patellar apex [41] (Table 1). Future research is expected to evaluate the suitability of advanced quantitative MRI techniques for evaluating inflammatory and destructive change in the JIA knee, including dynamic contrast-enhanced (DCE)-MRI, T2 mapping, T1 rho and diffusion-weighted imaging (DWI) [91–97]. Now, these advanced imaging techniques are used mainly in the setting of research and, to a lesser extent, in daily practice.

##### Point to consider

In children with a suspected inflammatory arthropathy and knee involvement, pre- and post-contrast MR images can be helpful for the evaluation of the degree of synovitis. To ensure accurate comparison between previous and present examinations, timing of post-contrast MR images should be standardised.

##### Rationale

Diagnostic accuracy of unenhanced MRI for evaluating knee synovitis is limited compared to contrast-enhanced MRI. However, the timing of post-contrast images strongly influences the enhancement, synovial thickness and total inflammation scores in the assessment of synovitis.

##### Controversies/future developments

**Fig. 6:**
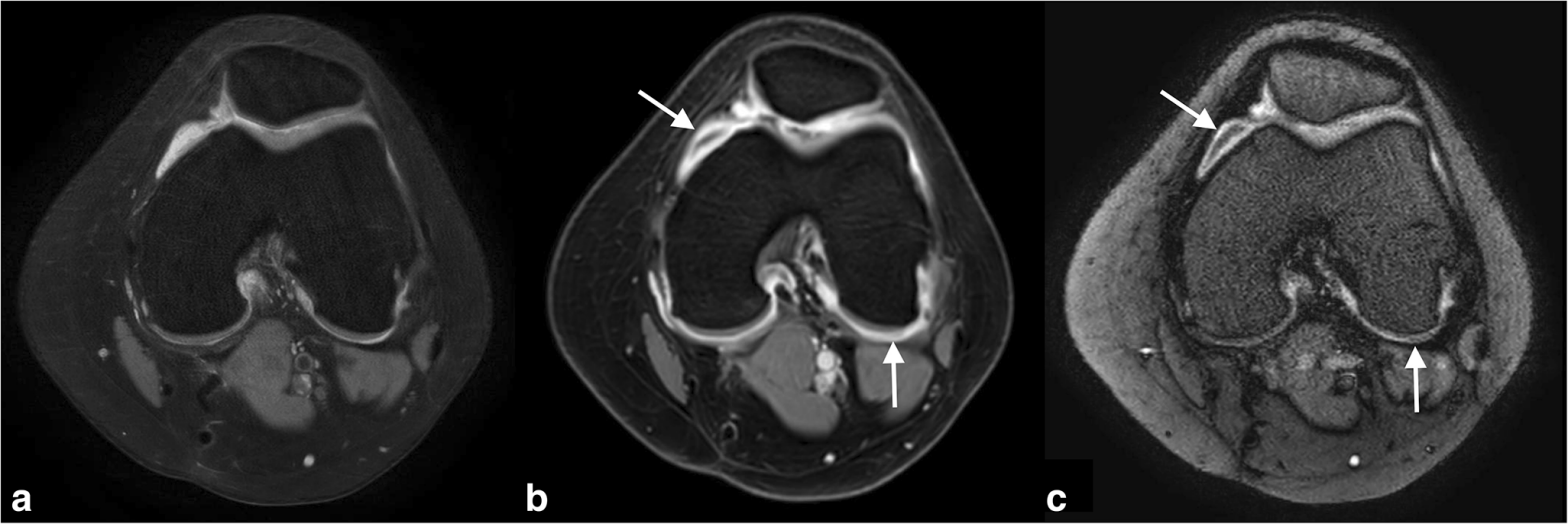
A 14-year-old boy with oligo-articular JIA and knee arthritis. MRI of the knee with (**a**) an axial T2-weighted fat-saturated image, (**b**) an axial T1-weigted fat-saturated post-Gd image showing an enhancing thickened synovial membrane retro-patellar (arrow) and posterior of the condyles (arrow) and (**c**) an axial double inversion recovery (DIR)–weighted Gd-free image showing a similar picture (arrows)

Should we aim for a broader clinical applicability of non-contrast–enhanced MRI techniques for the evaluation of knee synovitis, such as DWI and double inversion recovery imaging (Fig. 6)?

## Conclusion

In this article, we discussed paediatric-specific imaging characteristics of the most commonly involved and clinically important joints in JIA. Conventional radiographs have a lower sensitivity and specificity for disease activity, early arthritic disease detection and monitoring response to therapies, in addition to exposure to ionising radiation (Table 3). Nonetheless, radiography is valuable in the assessment of growth plates and epiphyses in the hand, to detect peri-articular osteoporosis in longer-standing JIA as well as spinal alignment.

**Table 3:** Summary

Joint	Conventional radiography^a^	Ultrasound	MRI
TMJ	Not indicated	Not indicated	For patients with clinical suspicion of TMJ JIA, fluid-sensitive, closed and open mouth views are suggested. Contrast-enhanced sequences are proposed since it can be helpful in evaluating synovial inflammation
Spine	For assessment of alignment, growth disturbances and bony changes Take care in interpretation of dynamic images	Not indicated	Consider contrast-enhanced MRI at baseline
SI joint	Not indicated	Not indicated	Consider MRI in children with suspected axial SpA. MRI could include SI joints and hips and consider screening MRI of the spine
Wrist	High-resolution radiographs of wrists and hands at diagnosis and follow-up, especially for evaluating growth disturbances	For the detection of joint effusion, synovitis and tenosynovitis Aiding joint injections	MRI for assessment of effusions and synovitis Structural abnormalities can be detected, but be aware of normal variants mimicking disease
Hip	At diagnosis to exclude other causes of joint pain, helps to evaluate growth disturbances and destructive change	For detection of effusion and synovitis and aiding joint injections	Consider MRI at baseline and at follow-up when ultrasound is equivocal
Knee	At diagnosis to exclude other causes of joint pain, helps to evaluate growth disturbances	For the assessment of joint effusions, synovitis and aiding joint injections	In children with a suspected inflammatory arthropathy and knee involvement, pre- and post-contrast MRI for evaluation of synovitis is suggested for the evaluation of the degree of synovitis. Structural abnormalities can be detected. Standardise timing after contrast for all imaging to ensure comparability is recommended

^a^The potential risks associated with exposure to ionising radiation must always be considered when using conventional radiography

Radiation protection is a priority in children with JIA; thus, in dedicated centres, the use of ultrasound or MRI in peripheral joints affected by JIA should be considered. Particularly in peripheral joints, ultrasound can be helpful for the assessment of inflammation, in differential diagnosis, and it can be useful for guiding joint injections. In JIA, MRI is the most validated technique for the assessment of inflammation and early destructive change. MRI could be of added value depending on local resources and expertise, but it should be considered as the modality of choice to assess the axial skeleton or where the clinical presentation overlaps with JIA, such as in osteomyelitis. Further imaging with radiographs and/or MRI should be guided by the preliminary findings, inconclusive US, atypical clinical presentation, chronic disease or when assessing response to therapy.

Finally, we have provided clinically applicable joint-specific points to consider on behalf of the ESSR arthritis subcommittee and the ESPR musculoskeletal imaging taskforce, highlighting areas of existing controversy/need for further study.

## Electronic supplementary material

ESM 1 (DOCX 35 kb)

## Abbreviations

ASAS: Assessment in Spondyloarthritis International Society
CARRA: Childhood Arthritis and Rheumatology Research Alliance
CT: Computed tomography
ERA: Enthesitis-related arthritis
ESPR: European Society of Paediatric Radiology
ESSR: European Society of Musculoskeletal Radiology
JAMRIS: Juvenile Arthritis MRI Scoring
JIA: Juvenile idiopathic arthritis
MRI: Magnetic resonance imaging
OMERACT: Outcome Measures in Rheumatology Clinical Trials
RAMRIS: Rheumatoid Arthritis MRI Scoring
SI: Sacroiliac
SpA: Spondyloarthritis
SPARCC: Spondyloarthritis Research Consortium of Canada
TMJ: Temporomandibular joint
US: Ultrasound
