# Supplementary material for: Imaging assessment of children presenting with suspected or known juvenile idiopathic arthritis: ESSR-ESPR points to consider
Source: Eur Radiol. 2020 May 12;30(10):5237–49. doi: 10.1007/s00330-020-06807-8 (PMC7476913; doi:10.1007/s00330-020-06807-8)
Supplement: Supplementary file 1 — (DOCX 35 kb) [file 330_2020_6807_MOESM1_ESM.docx]

*Imaging Assessment of Children Presenting with Suspected or Known Juvenile Idiopathic Arthritis: ESSR-ESPR points to consider*

**Supplementary File**

Examples of MRI protocols for specific JIA joints as recently published in literature.

***Temporomandibular joints***

Example of a consensus MRI protocol for the temporomandibular joint in JIA, as adapted from Miller et al. [1].

| **Sequence*** | **Acquisition order** | **Plane** | **Goal** | **Recommended or optional** |
| --- | --- | --- | --- | --- |
| T2-weighted FS or STIR | Interchangeable, pre Gd | Sagittal oblique | BME, effusion, synovial thickening, condylar flattening, erosions, disk abnormalities | Recommended |
| T1-weighted | Interchangeable, pre Gd | Sagittal oblique,  coronal or coronal oblique | BME, condylar flattening, erosions, disk abnormalities | Recommended |
| T1-weighted FS | Pre Gd | Sagittal oblique | Reference for joint enhancement | Optional |
| PD-weighted fast spin echo | Interchangeable, pre Gd | Sagittal oblique | Disk abnormalities | Optional |
| Gradient echo sequence | Interchangeable, pre Gd | Sagittal oblique | Condylar flattening, erosions | Optional |
| T1-weighted FS post-Gd | post Gd (first) | Sagittal oblique | Bone marrow and joint enhancement, condylar flattening, erosions, disk | Recommended |
| T1-weighted FS post-Gd | post Gd (second) | Coronal or coronal oblique | Bone marrow and joint enhancement, erosions, disk abnormalities | Recommended |
|  |  |  |  |  |
| * Maximum pixel size should be 1x1 mm, ideally 0.5x0,5 mm; maximum slice thickness of 3 mm, ideally 2 mm | | | | |
| FS: Fat saturated | | | | |
| BME: Bone marrow oedema | | | | |
| PD: Proton density | | | | |
| Gd: Gadolinium based contrast agent | | | | |

***Spine***

Example of MRI protocol for the cervical spine in JIA as suggested by the authors. There are no published MRI protocols.

Example of a consensus MRI protocol for the thoracolumbar spine in JIA, as adapted from an adult protocol from Sudol-Szopinska et al. [2]. There are no published consensus MRI protocols for children.

| **Sequence** | **Acquisition order** | **Plane** | **Goal** | **Recommended or optional** |
| --- | --- | --- | --- | --- |
| *Cervical spine* |  |  |  |  |
| T2-weighted FS or STIR | Interchangeable, pre Gd | Sagittal | BME, effusion, synovial thickening, erosions | Recommended |
| T2-weighted FS or STIR | Interchangeable, pre Gd | Axial^a^ or coronal | BME, effusion, synovial thickening, erosions | Recommended |
| T1-weighted | Interchangeable, pre Gd | Sagittal | Erosions, deformities, ankylosis | Recommended |
| T1-weighted FS post-Gd | post Gd | Axial^a^ or coronal | Bone marrow and joint enhancement, erosions | Recommended |
| T1-weighted FS post-Gd | post Gd | Sagittal | Bone marrow and joint enhancement, erosions | Recommended |
|  |  |  |  |  |
| *Thoracolumbar spine* |  |  |  |  |
| T1-weighted | Pre Gd | Sagittal | Erosions, deformities, ankylosis | Recommended |
| STIR/TIRM | Pre Gd | Sagittal | Disk abnormalities | Recommended |
| T2-weighted FS or STIR | Pre Gd | Axial | If optimal assessment of BME at posterior joints is needed | Optional |
| T1-weighted FS post-Gd | post Gd | Sagittal / axial | Bone marrow and joint enhancement, erosions | Optional |
|  |  |  |  |  |
|  | | | | |
| FS: Fat saturated | | | | |
| BME: Bone marrow oedema | | | | |
| STIR: short tau inversion recovery | | | | |
| Gd: Gadolinium based contrast agent | | | | |
| ^a^ Axial preferable | | | | |

***Sacroiliac joints***

Example of a consensus MRI protocol for the sacroiliac joints in JIA, as suggested by the authors, partly adapted from adult protocol from Sudol-Szopinska et al. [2]. There are no published consensus MRI protocols for sacroiliac joints for children.

| **Sequence** | **Acquisition order** | **Plane** | **Goal** | **Recommended or optional** |
| --- | --- | --- | --- | --- |
| T2-weighted FS or STIR | Interchangeable, pre Gd | Coronal oblique^a^ | BME, inflammation at site of erosion, capsulitis, enthesitis, joint space fluid | Recommended |
| T1-weighted | Interchangeable, pre Gd | Coronal oblique^a^ | Erosions, fat lesion, backfill, sclerosis, bony bud, ankylosis | Recommended |
| T2-weighted FS or STIR | Interchangeable, pre Gd | Axial^b^ | BME, inflammation at site of erosion, capsulitis, enthesitis, joint space fluid | Recommended |
| T1-weighted FS post-Gd | post Gd | Coronal oblique^a^ | Bone marrow and joint enhancement, erosions | Optional |
| T1-weighted FS post-Gd | post Gd | Axial^b^ | Bone marrow and joint enhancement, erosions | Optional |
|  |  |  |  |  |
|  | | | | |
| * Note: time permitting, field of view of axial images should include hip joints. A narrow field of view is preferred for coronal images to maximize detail at the SI joints. | | | | |
| FS: Fat saturated | | | | |
| BME: Bone marrow oedema | | | | |
| STIR: short tau inversion recovery | | | | |
| Gd: Gadolinium based contrast agent | | | | |
| ^a^ Coronal oblique: slice of sacroiliac joints in coronal plane relative to the tangent of the posterior surface of S2 vertebral body | | | | |
| ^b^ Axial: transverse slice of sacroiliac joints perpendicular to the coronal oblique slice | | | | |

***Wrist***

Example of a JIA MRI wrist protocol for children with a suspected inflammatory arthropathy and wrist involvement, as adapted from Avenarius et al. [3].

| **Sequence** | **Plane** | **Goal** | **Recommended or optional** |
| --- | --- | --- | --- |
| Cartilage specific | Coronal | ﻿Cartilage coverage bone erosions | ﻿Recommended |
|  | ﻿Multiplanar reconstruction | Cartilage coverage bone erosions | ﻿Optional |
| T2-weighted FS, STIR or T2 TSE (Dixon) | Coronal | ﻿Bone marrow oedema | ﻿Recommended |
|  | ﻿Axial | ﻿Effusion | ﻿Optional |
| T1-weighted TSE (Dixon) | Coronal | ﻿Anatomy | ﻿Recommended |
|  | ﻿Axial | Anatomy | ﻿Optional |
| T1-weighted TSE FS pre- /post-Gd | Coronal | ﻿(teno)synovitis and overall inflammation | Recommended |
|  | ﻿Axial | ﻿(teno)synovitis and overall inflammation | Recommended |
|  |  |  |  |
| FS: Fat saturated | | | |
| ﻿STIR: Short tau inversion recovery | | | |
| ﻿TSE: Turbo spin echo | | | |
| Gd: Gadolinium based contrast agent | | | |

***Hip***

Example of a ﻿suggested MRI protocol for the hips in children with suspected or established JIA, as adapted from Shelmerdine et al. [4].

| **Sequence** | **Plane** | **Goal** | **Recommended or optional** |
| --- | --- | --- | --- |
| T2-weighted FS or STIR | Coronal or axial | Joint effusion, bone marrow oedema, | Recommended |
| ﻿T1-weighted spin echo | Coronal | Bone marrow oedema, bone erosions | Recommended |
| ﻿T1-weighted spin echo FS | Coronal or axial | Synovial thickening, joint effusion | Optional |
| T1-weighted spin echo FS post-Gd | Coronal or axial | Synovial thickening, joint effusion | Recommended |
|  |  |  |  |
| FS: Fat saturated | | | |
| ﻿STIR: Short tau inversion recovery | | | |
| Gd: Gadolinium based contrast agent | | | |

***Knee***

Example of a JIA MRI knee protocol for children with a suspected inflammatory arthropathy and knee involvement, as adapted from Hemke et al. [5].

| **Sequence** | **Plane** | **Goal** | **Recommended or optional** |
| --- | --- | --- | --- |
| T2-weighted FS or STIR (mDixon)* | Sagittal | Joint effusion, BME, bone erosions | Recommended |
| T2-weighted FS or STIR (mDixon)* | Coronal | BME, bone erosions | Recommended |
| T1-weighted (mDixon)* | Coronal | BME, bone erosions | Recommended |
| Gradient echo / PD-weighted | Sagittal | Cartilage loss | Recommended |
| T1-weighted FS post-Gd | Axial | Synovial thickening, joint effusion | Recommended |
| Gradient echo (3D) | Axial | Cartilage loss | Optional |
| T1-weighted FS pre-Gd | Axial | Synovial thickening, joint effusion | Optional |
| T1-weighted FS post-Gd | Sagittal | Synovial thickening, joint effusion | Optional |
|  |  |  |  |
| * mDixon best option if available | | | |
| FS: Fat saturated | | | |
| BME: Bone marrow oedema | | | |
| PD: Proton density | | | |
| Gd: Gadolinium based contrast agent | | | |

***References***

1. Miller E, Inarejos Clemente EJ, Tzaribachev N, et al (2018) Imaging of temporomandibular joint abnormalities in juvenile idiopathic arthritis with a focus on developing a magnetic resonance imaging protocol. Pediatr Radiol 48:792–800. https://doi.org/10.1007/s00247-017-4005-8

2. Sudoł-Szopińska I, Jurik AG, Eshed I, et al (2015) Recommendations of the ESSR Arthritis Subcommittee for the Use of Magnetic Resonance Imaging in Musculoskeletal Rheumatic Diseases. Semin Musculoskelet Radiol 19:396–411. https://doi.org/10.1055/s-0035-1564696

3. Avenarius DFM, Nusman C, Malattia C, et al (2018) Current status of wrist imaging in juvenile idiopathic arthritis. Pediatr Radiol 48:801–810. https://doi.org/10.1007/s00247-017-4063-y

4. Shelmerdine SC, Di Paolo PL, Tanturri de Horatio L, et al (2018) Imaging of the hip in juvenile idiopathic arthritis. Pediatr Radiol 48:811–817. https://doi.org/10.1007/s00247-017-4022-7

5. Hemke R, Tzaribachev N, Barendregt AM, et al (2018) Imaging of the knee in juvenile idiopathic arthritis. Pediatr Radiol 48:818–827. https://doi.org/10.1007/s00247-017-4015-6
